# Surgical resection of soft tissue metastasis in cancers: A single‐center study of 77 cases over a 7‐year period

**DOI:** 10.1002/cam4.6808

**Published:** 2023-12-11

**Authors:** Qingrong Ye, Tu Hu, Zhengwang Sun, Peihang Xu, Chunmeng Wang, Yangbai Sun, Wangjun Yan

**Affiliations:** ^1^ Department of Musculoskeletal Surgery Fudan University Shanghai Cancer Center Shanghai China; ^2^ Department of Oncology Shanghai Medical College of Fudan University Shanghai China

**Keywords:** cancer of unknown primary, port site metastasis, soft tissue metastasis, survival analysis

## Abstract

**Introduction:**

Soft tissue metastasis (STM) of cancers, encompassing skeletal muscle and subcutaneous tissue metastasis, is less common due to unique homeostatic conditions. With longer life expectancy and the advent of new imaging modalities, clinical physicians will increasingly encounter and manage such cases. This study retrospectively reviewed cases of STM in visceral cancers who underwent surgery at Fudan University Shanghai Cancer Center over a 7‐year period.

**Methods:**

Data were collected through a comprehensive review of medical records, including demographic variables, primary tumor characteristics, surgical data, tumor pathology, and outcomes. Survival analysis was performed using Kaplan–Meier curves.

**Results:**

The study included 77 cases with a median follow‐up period of 854 days. The most common primary tumor sites were the lung (11) and breast (10). The abdominal wall was the most frequent site of metastasis. The combination of visceral metastasis, age over 52 years, and a history of primary tumor correlates with a poorer prognosis. Surgical‐related metastases are associated with a higher degree of differentiation. Additionally, we have identified a better prognosis for patients with cancer of unknown primary (CUP) exhibiting potential resectable soft tissue metastases.

**Conclusion:**

The combination of visceral metastasis, age over 52 years, and a history of primary tumor suggest a poorer prognosis. While no significant impact on survival was observed for patients with lymph node metastasis. Surgical‐related metastases are associated with a higher degree of differentiation. CUP patients with potentially resectable soft tissue metastases should be considered for surgical intervention.

## Graphical abstract

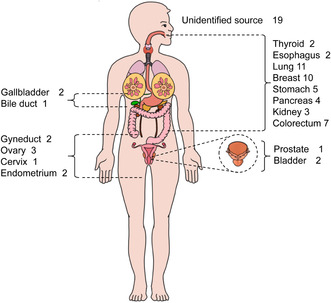

## 1 INTRODUCTION

Soft tissue metastasis (STM) of cancers, defined as metastasis to skeletal muscle and subcutaneous tissues, usually manifests as a soft tissue mass.^1^ Distinguishing STM from primary soft tissue tumors is essential, as their treatment and prognosis significantly differ.^2^ The prevalence of STM in autopsy studies ranges from 16%–17.5%.^3^, ^4^ Clinically, soft tissue metastases are relatively rare. However, due to a lack of reports, the clinical prevalence is difficult to ascertain. Glockner et al.^5^ analyzed 1421 patients who presented with a solitary soft‐tissue mass. The authors reported a frequency of metastases to soft tissue of 1.8%.

With the extension of cancer survival, there is an increasing trend in the incidence of soft tissue metastases (STS).^1^, ^6^ Furthermore, new imaging modalities, such as Positron Emission Tomography‐Computed Tomography (PET‐CT), have demonstrated higher sensitivity in detecting soft tissue metastases.^7^ As a result, orthopedic surgeons and other sub‐specialists, such as surgical oncologists, are increasingly encountering these cases and should be familiar with the principles of their management.

Surgery is one of the treatment modalities for STM, aiming at local control, pain relief, and prolonged survival.^8^ There is a need for more data to support detailed indications for surgical treatment and prognosis assessment as the prevalence increases.

Information in the literature regarding the clinical presentation, treatment, and oncological outcomes of cancer soft tissue metastases is limited, primarily reported in the form of case reports^9^, ^10^, ^11^, ^12^ and case series.^1^, ^2^, ^4^, ^13^, ^14^, ^15^ This is why there is still controversy surrounding the optimal therapeutic approach and expected oncological outcomes.

Our institution, Fudan University Shanghai Cancer Center (FUSCC), stands as one of the largest national comprehensive cancer centers in China and has extensive experience in STM treatment based on our multidisciplinary team (MDT). Annually, we provide care to approximately 30,000 patients with a wide range of soft tissue disorders.

The purpose of this study, therefore, is to (1) provide a descriptive analysis of the demographic characteristics of soft tissue metastases in our case series, (2) identify key prognostic factors associated with surgical intervention for soft tissue metastasis, and (3) provide further research information on the treatment approaches and oncological outcomes of STM.

## 2 MATERIALS AND METHODS

### 2.1 Patient selection

A retrospective analysis was conducted on cases of soft tissue mass resection at the Bone and Soft Tissue Tumor Surgery Center of FUSCC, covering the period from January 2016 to March 30, 2023. The aim was to identify patients with metastases of malignant tumors from visceral organs to soft tissues by reviewing their medical records.

#### 2.1.1 Inclusion criteria

1. Patients who underwent resection of the soft tissue mass.
2. Patients with a histological diagnosis of metastatic cancer confirmed through postoperative pathology.
3. Patients with comprehensive and complete medical records, including surgical data, imaging data, laboratory reports, and complete follow‐up records.

#### 2.1.2 Exclusion criteria

1. Patients with isolated lymph node metastasis only.
2. Patients with pathological reports or clinical diagnosis indicating sweat gland cancer, melanoma, hematological malignancies, or tumor originating from soft tissues.
3. Patients who only underwent biopsy.

### 2.2 Data collection

Retrospective data retrieval encompassed various aspects, including demographic variables, primary tumor characteristics and management, surgical data, tumor pathology, no‐surgery treatment, and outcomes. A prospectively maintained database was utilized for data extraction.

### 2.3 Follow‐up

Follow‐up was conducted through telephone and outpatient re‐examinations until the date of death or study cutoff (March 30, 2023). The initial postoperative outpatient visit occurred within the first 3 months after surgery, followed by annual outpatient follow‐up visits in the second to fifth year, and subsequently every 2 years. OS was computed from the date of metastasis resection to the date of death or the last follow‐up. If a patient's metastatic site underwent recurrent surgery, the calculation was based on the date of the first surgery performed at our institution.

### 2.4 Statistical analysis

Overall survival analysis results were performed using Kaplan–Meier plot graphics with Log Rank test (Mantel–Cox) and Gehan–Breslow–Wilcoxon test to evaluate significant differences. And Mann–Whitney *U* test and Chi‐Square Tests were utilized in analysis.

Univariate influences of prognostic factors on study endpoints were analyzed using log‐rank test. Due to the moderate size of the cohort, no multivariate analysis was performed. A significance level of *p* < 0.05 was considered statistically significant. The median duration of follow‐up was calculated using Schemper's method. The X‐Tile software was used to identify the optimal cutoff value for age.^16^ Analyses were conducted with R software, version 4.2.0 (http://www.R‐project.org) and SPSS Statistics 20, and graphs were conducted using GraphPad Prism 7.0.

## 3 RESULTS

### 3.1 Demographic characteristics

During the period from January 2017 to March 30, 2023, 149,360 patients underwent resection of soft‐tissue tumor. A total of 77 cases were included, categorized based on anatomic site, primary origin, age, and sex (see Table 1). The median follow‐up period was 854 days. Detailed information on resection margins, depth of metastasis, non‐surgical treatments, and local recurrence is presented in the Table S1.

**FIGURE 1:**
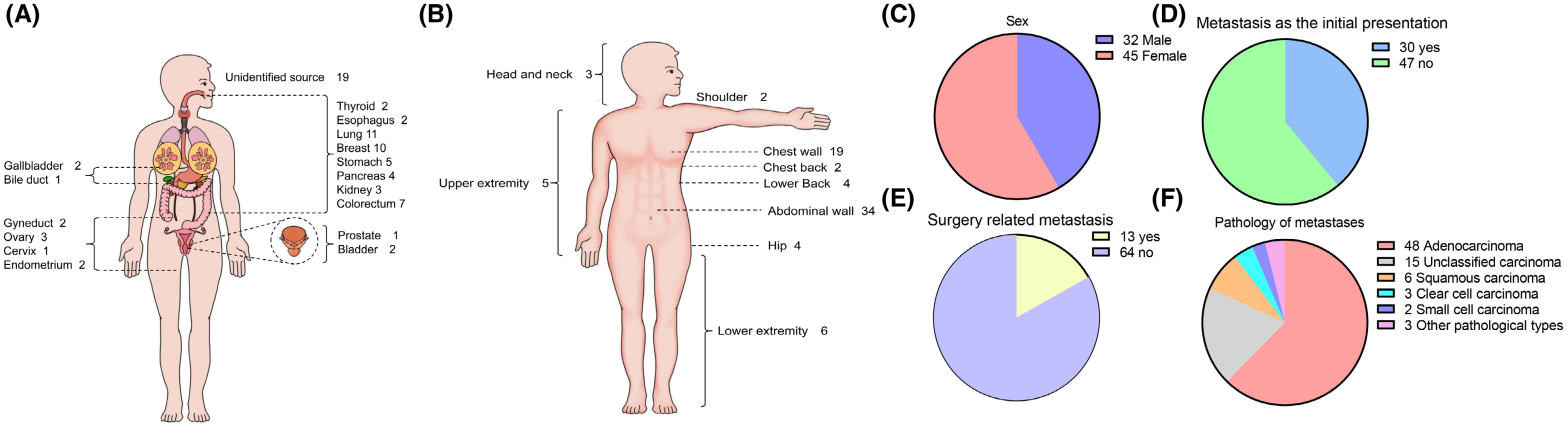
Characteristic of STM cases. (A) Site of primary tumor. (B) Site of soft tissue metastasis. (C) Distribution of gender. (D) Distribution of cases with metastasis as the initial presentation. (E) Distribution of surgery related metastasis. (F) Distribution of Pathology.

Among the patients, 45 were women and 32 were men. The most common site of the primary tumor was the lung (11 patients), followed by the breast (10 patients). The primary tumor was located in the cervix, bile duct, and prostate in one patient each. Additionally, 19 patients had an unknown primary origin (see Figure 1A). The most frequent sites of metastasis were the abdominal wall (33 cases), followed by the chest wall (18 cases), upper extremity (5 cases), hip (4 cases), lower extremity (4 cases), lower back (4 cases), head and neck (3 cases), shoulder (2 cases), and chest back (2 cases). Moreover, two patients exhibited metastasis in two different soft tissue sites: chest wall and lower extremity, and lower extremity and abdominal wall. (see Figure 1B).

Soft tissue metastasis manifested as the initial symptom of an occult tumor in 30 cases. It presented as a solitary late metastasis in 45 cases, as lymph node metastases in 15 cases, and as visceral metastasis in 17 cases. The occurrence of metastasis at the surgical incision site or port site metastasis (PSM) was classified as surgical‐related metastasis (SRM), which accounts for 16.9% (13 cases) (see Figure 1E, Table 1).

**TABLE 1:** Patient demographics and clinical data of 77 patients with soft tissue metastasis of visceral organ carcinoma.

	Overall (*n* = 77)
Sex (%)	
Female	45 (58.4)
Male	32 (41.6)
Age (mean (SD))	57.65 (12.99)
Primary tumor (%)	
Unidentified source	19 (24.7)
Lung	11 (14.3)
Breast	10 (13.0)
Colorectum	7 (9.1)
Stomach	5 (6.5)
Pancreas	4 (5.2)
Kidney	3 (3.9)
Ovary	3 (3.9)
Bladder	2 (2.6)
Endometrium	2 (2.6)
Esophagus	2 (2.6)
Gallbladder	2 (2.6)
Gyneduct	2 (2.6)
Thyroid	2 (2.6)
Bile ducts	1 (1.3)
Cervix	1 (1.3)
Prostate	1 (1.3)
Soft tissue site of metastasis (%)	
Abdominal wall	33 (42.9)
Chest wall	18 (23.4)
Upper extremity	5 (6.5)
Hip	4 (5.2)
Lower Back	4 (5.2)
Lower extremity	4 (5.2)
Head and neck	3 (3.9)
Chest back	2 (2.6)
Shoulders	2 (2.6)
Chest wall and lower extremity	1 (1.3)
Lower extremity and abdominal wall	1 (1.3)
Extent of metastasis (%)	
Only soft tissue metastasis	45 (58.4)
With lymph node metastasis	15 (19.5)
With visceral metastasis	17 (22.1)
Metastasis Pathology (%)	
Adenocarcinoma	48 (62.3)
Unclassified carcinoma	15 (19.5)
Squamous carcinoma	6 (7.8)
Clear cell carcinoma	3 (3.9)
Small cell carcinoma	2 (2.6)
Mucoepithelial carcinoma	1 (1.3)
Nephroblastoma	1 (1.3)
Neuroendocrine carcinoma	1 (1.3)
Surgery related metastasis (%)	
No	64 (83.1)
Yes	13 (16.9)
Metastasis as the initial presentation (%)	
No	47 (61.0)
Yes	30 (39.0)
Degree of differentiation (%)	
Not documented	30 (39.0)
Poorly differentiated	28 (36.4)
Moderately differentiated	13 (16.9)
Highly differentiated	6 (7.8)

### 3.2 Case presentation

**FIGURE 2:**
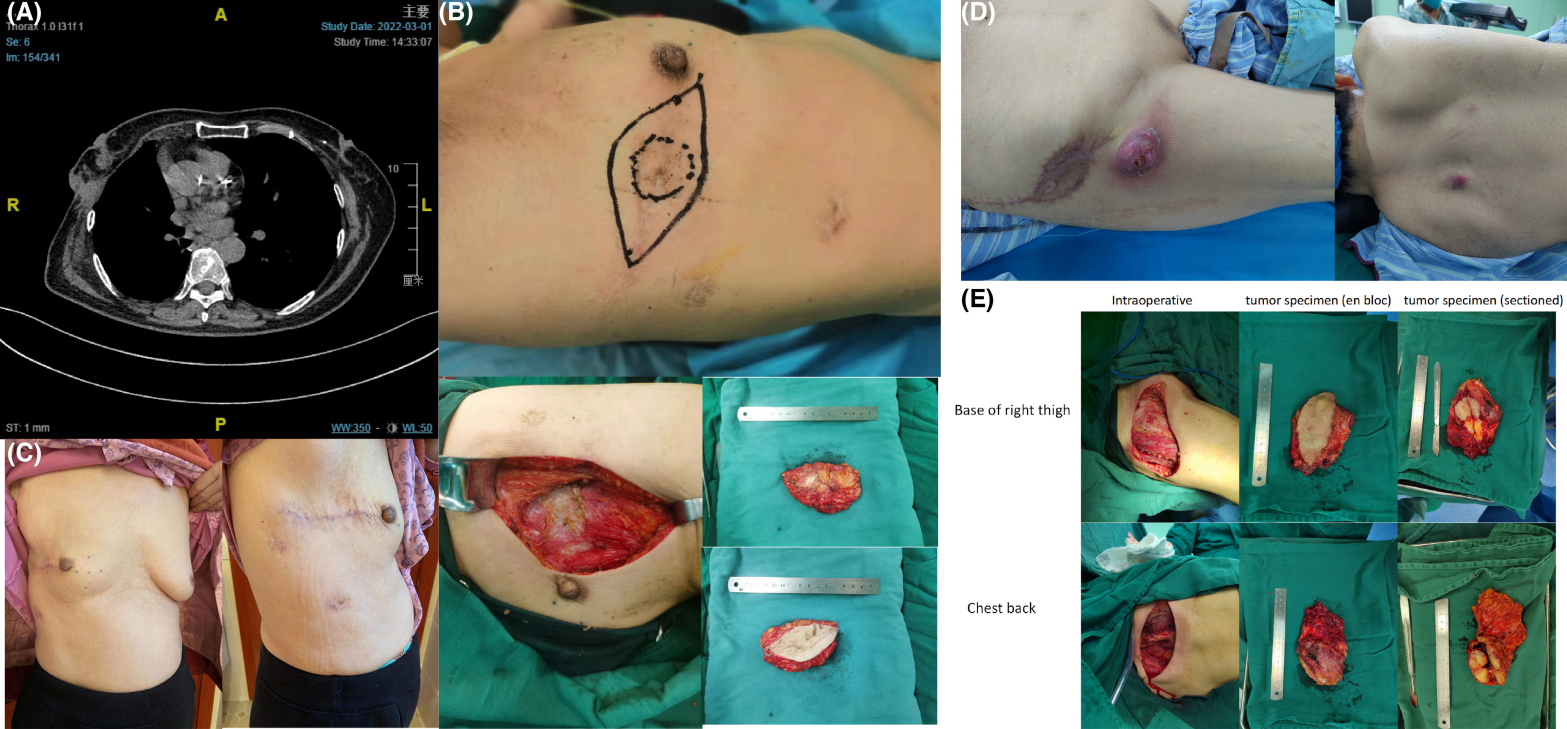
Case Presentation. (A) Case1: A 74‐year‐old female developed lung metastasis from colon cancer and experienced port‐site chest wall metastasis following thoracoscopic surgery. Preoperative CT scan indicates tumor in the right chest wall. (B) Preoperative, intraoperative photographs and surgical specimen. (C) March 2023, 1‐year postoperative follow‐up. (D) Case2: A 71‐year‐old female patient developed metastases from esophageal cancer in the thigh root and back. Preoperative photographs showing metastases at the chest back and thighs. (E) Intraoperative photographs showing tumor removal.

Figure 2 depicts two special cases of STM. (1): A 74‐year‐old female who underwent ascending colon cancer surgery in 2016, followed by chemotherapy. In 2017, she was diagnosed with lung metastasis and underwent video‐assisted thoracoscopic surgery for tumor resection, followed by postoperative radiotherapy. Pathological examination confirmed metastasis from colon cancer. In December 2019, a right‐sided chest wall mass was identified, suggestive of metastasis, leading to the performance of a chest wall tumor resection at an external hospital. In June 2020, a recurrence of the chest wall tumor was detected, accompanied by the presence of newly developed small nodules in the upper lobes of both lungs on CT scan, indicating potential metastasis. In September 2020, the patient underwent 60 Gy intensity‐modulated radiotherapy (IMRT) for the chest wall lesion as well as stereotactic radiotherapy (SRBT) for the lung lesions. The tumor size decreased following the radiotherapy. From November 2020 to January 2022, the patient received sequential treatments with capecitabine in combination with bevacizumab, fluorouracil, and oxaliplatin. In March 2022, the patient underwent chest wall tumor resection at our institution (Figure 2A,B). The postoperative pathological report confirmed the diagnosis of colorectal cancer origin. Following discharge, the patient received chemotherapy with oxaliplatin and underwent regular clinical monitoring. Currently, the lung lesions are controlled, with no evidence of tumor recurrence or progression within 1 year (Figure 2C). (2) A 71‐year‐old female with a history of esophageal cancer underwent esophagectomy and lymph node dissection in February 2020. Pathological examination revealed infiltration of the submucosal layer by poorly differentiated squamous cell carcinoma with a fungating growth pattern in the esophagus. Immunohistochemical analysis: EGFR+, ERCC++, P53+, CD34+. No lymph node metastasis was observed. No further treatment was administered postoperatively. In September 2022, a subcutaneous mass in the right iliac region was identified and subsequently excised at an external hospital. In October of the same year, a rapidly enlarging mass measuring 5 × 5 cm, protruding 3 cm above the skin surface, and accompanied by erythema and ulceration, was discovered at the root of the right thigh (Figure 2D). In November, a palpable mass was found on the right posterior chest wall (Figure 2D) associated with tenderness. The patient declined radiation and chemotherapy. In February 2023, the patient underwent surgical excision of the soft tissue mass at our institution (Figure 2E). Postoperative histopathological examination confirmed both poorly differentiated carcinoma consistent with metastatic esophageal cancer.

### 3.3 Overall survival (OS)

**FIGURE 3:**
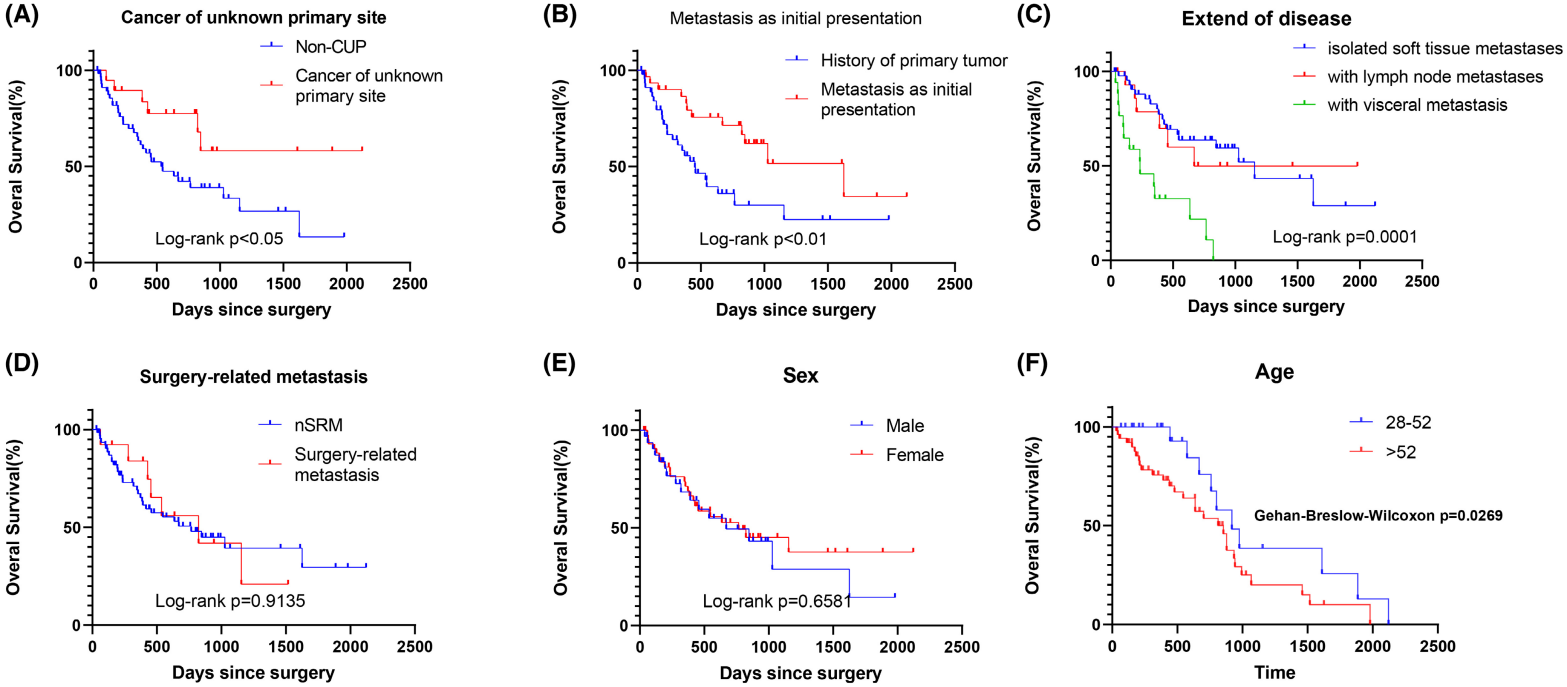
Comparison of OS between different clinical factors. (A) Overall survival between patients of cancer of unknown primary site non‐CUP. (B) Overall survival of whether metastasis is the initial presentation. (C) Overall survival between extend of disease. (D) Overall survival between whether surgery‐related metastasis. (E) Overall survival between sexes. (F) Overall survival between ages.

The median survival since STM resection was determined to be 766 days. Analysis using Kaplan–Meier survival plots indicated that patients with cancer of unknown primary site (CUP) had a significantly longer median survival time than those who had a confirmed primary site (*p* = 0.03, Figure 3A). Patients with metastasis as the initial presentation had a significantly longer median survival time than those who have Tumor history (*p* < 0.01, Figure 3B). And OS analysis according to extend of disease showed a significantly shorter median survival time in patients with widespread disease involving visceral metastasis (234 days, *p* = 0.0001; Figure 3C).

Overall survival analysis according to gender and surgery‐related metastasis indicated no statistically significant difference (*p* > 0.05, Figure 3D,E). With the X‐Tile software identifying the optimal cutoff value for age as 52, Gehan–Breslow–Wilcoxon test indicates statistical differences between the prognosis of patients with different age subgroups (*p* = 0.0269; Gehan–Breslow–Wilcoxon test, Figure 3F).

### 3.4 Surgery‐related metastasis

The degree of differentiation was classified into four groups based on pathological reports: poorly differentiated (28), moderately differentiated (13), highly differentiated (6), and not documented (30). After excluding the not‐documented group, Mann–Whitney *U* test was employed for analysis.

Surgery‐related metastases exhibited a higher Degree of differentiation (Mann–Whitney *U* test: *U* = 79, *Z* = −2.491, *p* = 0.013).

### 3.5 Resection margins

**FIGURE 4:**
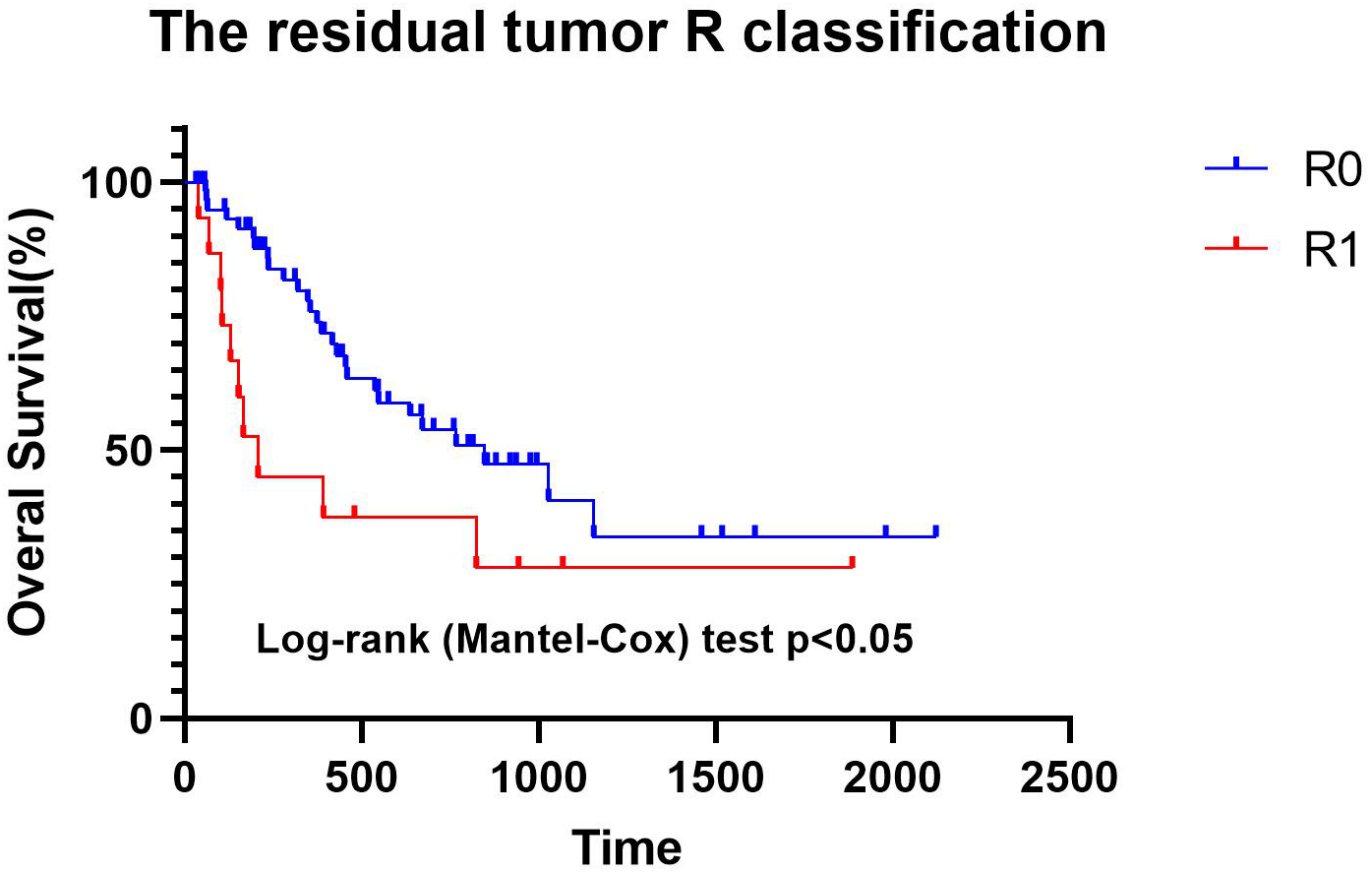
Overall survival according to the R classification.

The residual tumor (R) classification includes R0 (61 cases)—no residual tumor; R1 (15 cases)—microscopic residual tumor; and Rx (1 case)—pathology report did not describe the surgical margin. Overall survival analysis based on the R classification revealed a significantly shorter median survival time in patients with microscopic residual tumors (Figure 4, 206 days, log‐rank (Mantel–Cox) test *p* < 0.05). The chi‐square test indicated a significant correlation between the R classification and the local recurrence rate (*χ* ^2^ = 9.837, *p* < 0.002).

## 4 DISCUSSION

Despite soft tissue representing more than 50% of total body mass and receiving a large portion of total cardiac output, metastasis to soft tissue is very uncommon.^2^, ^11^ Various theories have been proposed to account for the relative paucity, including constant cellular trauma resulting from muscle contraction, heightened permeability of tumor cells, specific homeostatic factors, and pH fluctuations that impede tumor cell growth.^15^, ^17^

In this study, a retrospective analysis was conducted on patients who underwent surgical excision of soft tissue masses over 7 years at a large cancer center, identifying a total of 77 patients with visceral malignant soft tissue metastases. Our screening criteria excluded cases with isolated lymph node involvement, leading to a lower incidence of soft tissue metastases in areas with extensive lymphatic drainage, such as the axilla or groin. Additionally, we excluded soft tissue sarcoma (STS) by thoroughly examining pathology reports. Metastatic cancers of cutaneous squamous‐cell carcinoma are also excluded from the analysis due to substantial differences in prognosis and treatment compared to primary tumors originating from visceral organs.^18^ However, among metastasis with unknown primary origin, squamous cell skin carcinomas may still be present and need to be considered during differential diagnosis.

The most commonly reported malignancies in the literature to result in distant soft tissue metastases are lung, kidney, and colon carcinoma.^6^, ^13^, ^19^, ^20^, ^21^, ^22^ Our findings indicate that lung cancer, breast cancer, and colorectal cancer are the most common primary malignant tumors, in that order. In contrast, there were only three cases of kidney cancer. The increase in the proportion of breast cancer and the decrease in the proportion of kidney cancer may be attributed, on the one hand, to changes in the disease spectrum in recent years and, on the other hand, to the varying surgical indications for different cancer types with soft tissue metastasis.^23^

The most common histologic diagnosis reported in the literature on distant soft tissue metastases is adenocarcinoma, followed by renal cell carcinoma of clear cell type and squamous cell carcinoma, but many other histologic types have also been reported.^2^, ^24^, ^25^ And metastasis is more frequently observed in the axial region of the body compared to the extremities.^20^ In our study, adenocarcinoma is the most common histological subtype. And we also identified cases of squamous cell carcinoma, small cell carcinoma, and other types. Also, there were 66 metastases in the axial region, and 9 in the limbs except 2 cases where the STM involved both the trunk and limbs. This may suggest an affinity of metastases to muscles of this region. Out of the 11 cases involving limb metastasis, 5 cases (45.5%) were attributed to lung cancer.

A soft‐tissue mass that is caused by metastatic carcinoma could be easily misdiagnosed as a soft‐tissue sarcoma with physical examination and imaging studies, and their differentiation is important because the treatment and prognosis are markedly different.^6^ Magnetic resonance imaging (MRI) typically stands out as the preferred imaging modality in assessing soft‐tissue masses due to its exceptional tissue contrast and capacity to illustrate intricate anatomical features. Its capability to determine the extent of tissue engagement and the proximity of tumors to adjacent neurovascular system, tendons, or bones is crucial in shaping surgical or radiation treatment strategies.^1^ PET‐CT has shown higher sensitivity in detecting skeletal muscle metastasis, and, in many cases, these are subclinical lesions.^26^ However, Imaging findings in muscle metastases are nonspecific and are essentially indistinguishable from primary soft‐tissue sarcomas. A definitive diagnosis in the vast majority of cases, is done through core needle or open biopsies.

In the reported case series, the majority of distant soft tissue metastases presented as symptoms of previously diagnosed malignant tumors. While a significant portion of these cases also manifested as the first presentation of occult malignancies.^6^ Among our cases, soft tissue metastasis was the initial presentation of the disease in 30 cases (39%), with 11 of them subsequently discovering the primary tumor elsewhere. In the other 19 cases, the primary origin remained undetermined until the last follow‐up or death, despite undergoing standard assessments and imaging studies. These cases were classified as cancers of unknown primary (CUP).^27^ Here, based on the known data, we emphasize that STM cannot be simply considered as a terminal manifestation of malignancy. Regardless of whether there is a clinical history of other known primary cancers, STM should not be considered within the initial differential diagnosis.

Cancers of unknown primary accounts for 2%–5% of all diagnosed cancers worldwide and is characterized by early dissemination without a detectable primary tumor and an aggressive clinical course.^28^ Generally, CUP is one of the most fatal cancers with a median survival of about 3 months based on population‐based data.^29^ Even if the general prognosis remains poor, some clinicopathological characteristics can identify subgroups with more favorable outcomes based on pathology, clinical manifestations, organ involvement, and even genomic sequencing results, each with different prognoses. Approximately 20% of CUP cases belong to a favorable subset,^30^ whose subset includes patients with a potentially resectable tumor. Kaplan–Meier analysis in our cohort indicates statistical differences in prognosis between patients of CUP had a better and those with known primary tumors (*p* = 0.0154). One reason is that the subgroup of poor‐prognosis CUP cases often experience death within 3 months of diagnosis and are unable to be referred to our center. This finding indicates that having an unknown primary tumor can even be a favorable prognostic factor for survival in potentially resectable STMs. And this reinforces the viewpoint that soft tissue, as a specific site of metastasis in CUP, exhibits a relatively low site‐specific mortality rate.^28^ This highlights the importance of addressing the treatment needs of this subset of patients, despite their relatively small numbers and occurrence rates. Timely intervention for these patients is crucial, as it directly impacts their overall survival benefits. Despite the lack of data on unoperated CUP cases, which makes it challenging to compare the survival benefits of local surgical intervention, these results are encouraging. Further exploration of the value of surgical intervention in the treatment of this particular subgroup of CUP with a good prognosis is warranted.

Surgical resection of isolated soft tissue metastases is generally associated with a favorable prognosis, while systemic therapy or palliative surgery is often recommended for patients with extensive metastases to alleviate symptoms.^2^, ^14^ Therefore, we categorized patients into three groups based on the extent of metastasis: isolated soft tissue metastases, lymph node metastases, and visceral metastases. Our results showed no significant difference in prognosis between patients with isolated soft tissue metastases and those with lymph node metastases. However, patients with visceral metastases exhibited significantly shorter survival compared to the former two groups.

Although extensive disease is generally considered an adverse prognostic factor for any type of metastatic condition, our findings provide further data support in this regard. Furthermore, when selecting treatment strategies for STM patients with lymph node metastases, a more proactive consideration of wide local excision may be warranted. These patients may potentially achieve similar prognoses as those with isolated metastases.

As a strong predictor of prognosis. The prognostic significance of R classification is demonstrated by respective data for non‐small cell lung carcinoma, squamous cell carcinoma of esophagus, gastric carcinoma, ductal adenocarcinoma of the pancreas, colorectal carcinoma, and lung and liver metastases.^31^ Pretell‐Mazzini et al. reported an overall local recurrence rate of 8.7% in STM patients with microscopic residual tumors.^6^

We approach the impact of R classification on survival outcomes cautiously, as some patients in the R1 category underwent non‐wide local excision as palliative procedures, potentially with concurrent visceral metastasis. However, the records did not specify whether the surgeries were curative or palliative. Conversely, the shorter median survival observed in R1 patients correlates with higher rates of local recurrence, suggesting a significant influence of R classification on local recurrence.

An increasing body of clinical and experimental evidence suggests that invasive surgical procedures like tumor resection and biopsy might foster tumor progression and metastatic disease.^23^ Local effects of surgery include tumor seeding and the activation of a wound healing response.^32^ Additionally, surgery‐induced local and systemic immunosuppression can hinder the body's antitumor immune response and facilitate tumor cell survival.^33^, ^34^ Our study adopts a broad definition of soft tissue metastasis, encompassing hematogenous metastases to subcutaneous tissue and muscle, direct extension of visceral tumors to soft tissue, and transplant metastases in surgical incisions or port site metastasis (PSM). This approach enables us to potentially compare these Surgical‐Related Metastases (SRM) with other STM in terms of their clinical and oncological characteristics.

A total of 13 SRMs were identified in this study, with 12 cases originating from abdominal surgery and 1 case from thoracoscopic surgery (Figure 2A–C). Kaplan–Meier analysis did not indicate a significant difference in survival between patients with and without surgery‐related metastasis (Figure 2D). Considering the result arising from the small sample size and potential confounding factors, we further analyze differentiation degree information from pathological reports, excluding those lacking clear documentation of differentiation degree. The results revealed an association between surgery‐related metastases and a higher differentiation (Mann–Whitney *U* test: *U* = 79, *Z* = −2.491, *p* = 0.013). Therefore, we suggest that surgical factors may prompt the occurrence of malignant tumor metastasis before hematogenous dissemination, leading to an earlier differentiation level, which could potentially influence prognosis.

Carcinoma of the gallbladder that is suspected during cholecystectomy or identified through histological examination is referred to as incidental gallbladder carcinoma (IGBC).^35^ The incidence of gallbladder cancer diagnosed during or after a LC has been reported to range between 0.19% and 3.3% at various medical centers.^36^

Among these cases, six patients developed metastasis from PSM following laparoscopic cholecystectomy (LC) with an average age of 59.3 years, and the median survival was 638.5 days. Among the six patients, five were female. In two of these cases, LC was performed for presumed benign conditions, but gallbladder cancer was discovered postoperatively. In the remaining four cases, the absence of gallbladder pathology results or benign gallbladder specimen pathology, combined with metastatic lesion pathology and PET‐CT findings, hindered confirmation of the primary tumor origin.

It is important to acknowledge the limitations and deficiencies of our study. Firstly, all pathological data were obtained from surgical files, which may not fully reflect the oncologic characteristics and baseline of all patients with soft tissue metastases. Non‐surgical treatments information is available in the Table S1, but was not introduced into analysis due to confounding bias. Additionally, the specific surgical purpose (radical or palliative) was not accurately documented in the files. Moreover, our study covered a 7‐year period, and the follow‐up period may be insufficient to capture long‐term outcomes.

In conclusion, this is one of the largest series of surgical section soft tissue metastasis (*n* = 77) reported in the literature. In surgically excised soft tissue metastases, the combination of visceral metastasis, age over 52 years, and a history of primary tumor correlates with a poorer prognosis. Surgical‐related metastases are associated with a higher degree of differentiation. Additionally, we have identified a better prognosis for patients with Cancer of Unknown Primary (CUP) exhibiting potential resectable soft tissue metastases. Our study provides reference material for orthopedic and oncologic surgeons in clinical treatment and prognostic assessment, offering additional clinical and oncological evidence for this rare condition. Further studies are needed to identify biological factors that predispose patients with cancer to develop soft tissue metastases.

## AUTHOR CONTRIBUTIONS

**Qingrong Ye:** Data curation (equal); visualization (equal); writing – original draft (lead). **Tu Hu:** Data curation (equal); investigation (equal); methodology (equal); visualization (equal). **Zhengwang Sun:** Supervision (equal); validation (equal). **Peihang Xu:** Conceptualization (equal); software (equal). **Chunmeng Wang:** Formal analysis (equal); supervision (equal). **Yangbai Sun:** Conceptualization (lead); project administration (lead); writing – review and editing (lead). **Wangjun Yan:** Funding acquisition (lead); resources (lead); supervision (lead).

## FUNDING INFORMATION

This study was supported by grants from the National Natural Science Foundation of China (Grant No: 82072972 recipient: Wangjun Yan), and grants from the Shanghai Municipal Health Commission Research Project (#20194Y0242; recipient: Yangbai Sun).

## CONFLICT OF INTEREST STATEMENT

The authors declare that they have no competing interests.

## ETHICS STATEMENT

This study was approved by the Ethics Committee of Fudan University Shanghai Cancer Center, Shanghai, 200032, China. Each patient signed an informed consent document during the preoperative conversation. Written informed consent was obtained from the individual(s) for the publication of any potentially identifiable images or data included in this article.

## Supporting information


Table S1.

## Data Availability

The raw data is will be made available by the authors, without undue reservation. Further information can be available from the corresponding authors upon appropriate request.
